# The complete mitochondrial genome of the economic red alga, *Gracilaria chilensis*

**DOI:** 10.1080/23802359.2017.1390416

**Published:** 2017-10-17

**Authors:** Na Liu, Guoliang Wang, Yue Li, Lei Zhang, Maria Dyah Nur Meinita, Weizhou Chen, Tao Liu, Shan Chi

**Affiliations:** a College of Marine Life Sciences, Ocean University of China, Qingdao, PR China;; b CAS Key Laboratory of Genome Sciences and Information, Beijing Institute of Genomics, Chinese Academy of Sciences, Beijing, PR China;; c University of Chinese Academy of Sciences, Beijing, PR China;; d Faculty of Fisheries and Marine Science, Jenderal Soedirman University, Purwokerto, Indonesia;; e Marine Biology Institute, Shantou University, Shantou, PR China;; f Qingdao Haida BlueTek Biotechnology Co., LTD, Qingdao, PR China

**Keywords:** *Gracilaria chilensis*, complete mitogenome, stem loop, phylogenetic analysis

## Abstract

*Gracilaria chilensis* is an economically important marine alga. In this study, we obtained complete mitogenome of *G. chilensis* by high-throughput sequencing, which was mapped as a circular molecule of 26,897 bp with 27.56% GC content and was identified 53 genes, including 25 protein-coding genes, 2 rRNA genes, 26 tRNA genes, and 1 group II intron inserted into the *trnI* gene. In addition, a 162-bp stable stem loop was found in intergenic regions, which was most likely associated with DNA transcription and replication. The Bayesian phylogenetic tree of Gracilariaceae revealed that *G. chilensis* and *G. salicornia* and *G. changii* shared a closer relationship than *G. vermiculophylla* in the genus *Gracilaria.*

*Gracilaria chilensis* (Gracilariales, Rhodophyta) is one of the most important economic macroalgae (Troell et al. 1997). It is rich in agar and minerals, mainly used as a high-quality raw material for extracting agar and algin (Santelices and Doty 1989; Lobban and Harrison 1994). In China, it is mostly located in the South China Sea and the East China Sea, and less so in the Yellow Sea (Pan and Li 2010). At present, eight species’ mitogenomes of Gracilariaceae have been published on the NCBI sequence database, the size of mitogenomes ranges from 25 to 27 kb, with an average GC content of 27.92%.

Here, we sequenced *G. chilensis* which collected from Rongcheng, Shandong Province, China on July 21, 2016 (accession number: 2016070050, deposited in the Culture Collection of Seaweed at the Ocean University of China). Total DNA was extracted by the modified CTAB method (Doyle and Doyle 1990). Whole genome was performed by the Illumina HiSeq platform and four libraries were constructed. Approximately 35 contigs were used to assemble the complete mitogenome by aligning the available mtgenome sequence of published *G. salicornia* (NC_023784) as reference. The multiple protein and ribosomal RNA (rRNA) gene alignments were determined by BLAST similarity searches on the NCBI sequence database and compared to the published mitogenome of *G. salicornia.* tRNAscan-SE version 1.21 software (The Lowe Lab, Santa Cruz, CA) was employed to search for transfer RNA (tRNA) genes (Lowe and Eddy 1997).

The complete mitogenome of *G. chilensis* was 26,897 bp with an overall GC content of 27.56%, the nucleotide composition was 34.65% A, 14.22% C, 13.34% G, and 37.80% T (GenBank accession number MF401962). We identified 53 genes, including 25 protein-coding genes that spanned 18,126 bp, accounting for 67.4% of the mitogenome, 2 rRNA genes, and 26 tRNA genes with one group II intron interrupting the *trnI* gene. 4.67% of the mitochondrial DNA was non-coding. Most protein-encoding genes were encoded on the H-strand and tRNA genes evenly encoded on both H-strand and L-strand. All protein-encoding genes started with an ATG codon, and terminated with TAA and TAG codons. Mitogenome used a modified code in red algae, and TGA codon supposedly encoded for tryptophan (W), except in *Cyanidioschyzon merolae*. The genes present in the mitochondrial genome of *G. chilensis* were consistent with the mitogenome of Gracilariaceae. Additionally, we identified a 162-bp stable stem loop in the intergenic regions between *orf148* and *secY*, which was believed to be involved in DNA transcription and replication. The secondary structure was an inverted repeat at the demarcation point of transcription.

A total of 24 protein-encoding genes shared among the eight species of Gracilariaceae for which there are mitogenomic data were used to compute a Bayesian phylogenetic tree using MrBayes version 3.1.2 software (Software Foundation, Inc., Cambridge, MA; Ronquist and Huelsenbeck 2003). *Schizymenia duby* was used as the out-group. The eight species of Gracilariaceae were mainly divided into two branches (Figure 1). On the one hand, four species of the genus *Gracilaria* were clustered together, with *G. chilensis* and *G. salicornia* and *G. changii* sharing higher homology and thus a closer relationship than *G. vermiculophylla*. On the other hand, although *Gracilariopsis* species were included in the other branch, *G. lemaneiformis* and *G. chorda* were clustered into same class because mitogenome homology of their mitogenomes was 99.82% (Yang et al. 2014). Nevertheless, *G. andersonii* and *G. oryzoides* were clustered into another branch because they are parasitic and their mitogenomes were highly conserved.

**Figure 1.**
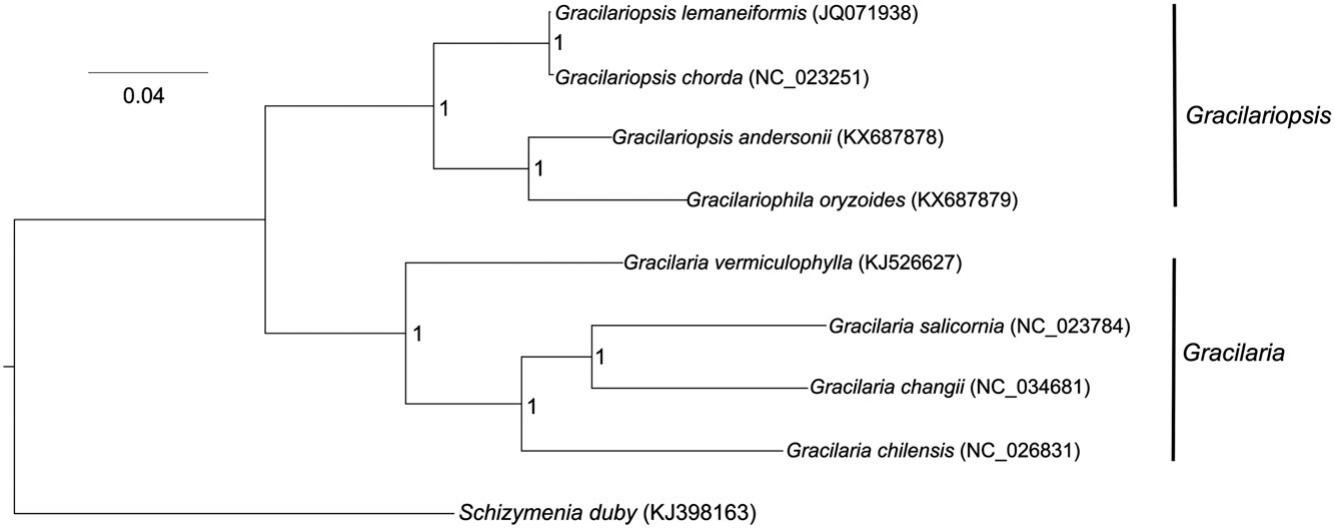
The phylogenetic tree was constructed based on common protein genes from eight species of Gracilariaceae available at the GenBank database. The posterior probability value was calculated on the basis of the Metropolis–Hastings-Green algorithm through four independent chains running for over 500,000 cycles until the *p* value was less than .01 and 25% aging samples were discarded. The posterior probabilities are shown at the nodes.
